# Blind footballers direct their head towards an approaching ball during ball trapping

**DOI:** 10.1038/s41598-020-77049-3

**Published:** 2020-11-20

**Authors:** Takumi Mieda, Masahiro Kokubu

**Affiliations:** 1 Graduate School of Comprehensive Human Sciences, University of Tsukuba, 1-1-1 Tennodai, Tsukuba, Ibaraki 305-8574 Japan; 2 Faculty of Health and Sport Sciences, University of Tsukuba, 1-1-1 Tennodai, Tsukuba, Ibaraki 305-8574 Japan

**Keywords:** Auditory system, Human behaviour, Perception

## Abstract

In blind football, players predict the sound location of a ball to underpin the success of ball trapping. It is currently unknown whether blind footballers use head movements as a strategy for trapping a moving ball. This study investigated characteristics of head rotations in blind footballers during ball trapping compared to sighted nonathletes. Participants performed trapping an approaching ball using their right foot. Head and trunk rotation angles in the sagittal plane, and head rotation angles in the horizontal plane were measured during ball trapping. The blind footballers showed a larger downward head rotation angle, as well as higher performance at the time of ball trapping than did the sighted nonathletes. However, no significant differences between the groups were found with regards to the horizontal head rotation angle and the downward trunk rotation angle. The blind footballers consistently showed a larger relative angle of downward head rotation from an early time point after ball launching to the moment of ball trapping. These results suggest that blind footballers couple downward head rotation with the movement of an approaching ball, to ensure that the ball is kept in a consistent egocentric direction relative to the head throughout ball trapping.

## Introduction

Blind football is designed to enable blind and partially sighted individuals to take part in the sport. Players localize the positions of the ball, teammates, and opponents by listening to sounds during games and daily training. The ball contains a sound system that causes it to make a noise when it bounces and rolls^1^, which enables players to localize the changeable position, as well as the initial position. In ball trapping, players need to predict the trajectory of the upcoming ball and intercept the ball corresponding to the position. Therefore, accurate localization of the ball using auditory information is necessary to underpin the success of ball trapping.

Previous studies have indicated that blind footballers accurately localize sound direction using auditory cues that humans are known to benefit from when localizing a sound^2^. For example, blind footballers are more precise than blind and sighted individuals in identifying the direction of sounds emitted from static loudspeakers^3,4^. Velten et al. also reported that blind footballers showed less-frequent front–back confusion^4^—that is, when a stimulus in front of a subject is localized to the rear, or vice versa^5^—which occurs due to the so-called “cone of confusion”^6,7^. A more recent study by Mieda et al.^8^ revealed that blind footballers had faster choice reaction times, but not simple reaction times, compared to sighted footballers when executing a stepping response towards the perceived direction of the static loudspeakers. They suggest that blind footballers identify sound direction more rapidly based on auditory cues, while also maintaining accuracy, compared to sighted footballers and nonathletes. Based on these findings, it is reasonable to assume that rapid and accurate identification of sound direction with auditory cues represents a strategy of blind footballers. However, to our knowledge, there has been no study on the strategy of blind footballers to identify the direction of a dynamic sound source, such as a moving ball, which is necessary to perform ball trapping.

Two aspects are important to increase the accuracy of sound localization. The first is to move the head; it is well known that head movements increase the accuracy of sound localization (i.e., identification of sound direction) when listeners are allowed to move their head during sound presentation. Previous studies have reported that head movements can reduce front–back confusion^9–11^, and improve accuracy of sound localization in the horizontal^9,12^ and elevation^13,14^ planes. The effect of head movements on sound localization has been studied previously by identifying the direction of the static sound source. Even in the case of localizing moving sound sources, some studies^15^ have reported that head movements also improve the accuracy of sound localization (i.e., smaller moving minimum audible angle thresholds) when listeners moved their head while audio signals were moved, compared to when listeners remained still during the audio signal motion. Taken together, head movements appear to be important in ball trapping, which requires the accurate identification of the direction of a dynamic sound source (i.e., a moving ball). Here, we investigate whether blind footballers use horizontal and vertical head movements to underpin the success of ball trapping.

The second aspect is to direct the head towards the sound source, which is necessary for accurate localization^16^. Previous studies have reported that sound localization is particularly accurate when the sound is in front of the listeners’ heads^17–19^. For instance, Makous & Middlebrooks^19^ showed that the size of errors and response variability were smallest for sounds presented in front of the listeners’ heads, while they were increased when the sounds were presented at more peripheral stimulus locations. Therefore, it is important for sound to be localized in front of the head using head rotation when localizing sounds from peripheral locations.

Auditory–motor performance, such as reaching and pointing to auditory targets, is also influenced by the location of a sound source in relation to the listeners’ heads. For instance, pointing error to auditory targets has been found to be smaller at the height of the chest than at the height of the feet^20^. Furthermore, higher accuracy of pointing to auditory targets was observed under the condition in which listeners showed a larger heading angle to the targets, as well as with a larger amplitude of head movements^21^. The authors suggest that the accuracy of pointing to auditory stimuli could be due to the contribution of heading towards the target providing a more accurate frame of reference for the anticipated control of pointing. Collectively, it is predicted that the direction of the auditory stimulus relative to the position of the head could affect the performance of auditory–motor tasks in blind footballers.

For other sports, such as cricket^22^ and baseball^23–25^, skilled batters have been shown to couple their head direction to the movement of the ball, known as a “head-ball coupling” strategy, in order to track the target. For instance, elite batters demonstrated a head-ball coupling movement when the ball direction was kept in a constant direction corresponding to the head in real batting^22,24,25^. Crucially, Mann et al.^22^ indicated an advantage to keep the target in a consistent direction relative to the head, because visuo–motor tasks, such as catching and hitting, are controlled in an egocentric manner. Based on the previous findings showing that sound location was mainly coded in head-centred reference frame^26,27^, it is likely to be important to track the ball in a consistent egocentric direction relative to the head, even in the case of auditory–motor tasks such as ball trapping. Thus, it is reasonable to expect that blind footballers would utilize a strategy to couple the head direction relative to the movement of the ball during trapping.

Finocchietti et al.^28^ studied the movement patterns of blind footballers compared to sighted footballers in a fast and repetitive ball passing task against a wall. The authors found that the blind footballers presented a greater trunk flexion angle at the lumbar (L1/T12) and thoracic (T9/T8) levels, but not at the cervical (T1/C7) level, when compared to those of sighted individuals while performing passes. They imply that forward leaning of the trunk seems to be a strategy related to maximizing the quality of auditory inputs to guide postural control during passing/receiving the ball. However, they did not examine movement patterns, such as the head and trunk, to localize the ball position during receiving, because the study was designed to analyse movement patterns immediately before and after kicking the ball. To clarify this issue, we investigated whether downward head rotation or forward tilt of the trunk could contribute to accurate sound localization for ball trapping.

The purpose of the present study was to determine the strategy of blind footballers to localize a dynamic sound source, such as a moving ball. Specifically, we investigated the characteristics of head rotations in blind footballers compared to sighted nonathletes when trapping an approaching ball. We hypothesized that blind footballers utilize larger head rotations in order to accurately perform ball trapping.

## Methods

### Participants

Participants were blind male footballers (n = 6; mean age, 27.3 ± 6.8 years; playing experience, 7.4 ± 3.5 years) and age-matched healthy sighted nonathletes (n = 6; mean age, 28.0 ± 5.2 years; no regular blind football training). This study adopted its experimental design, comparing blind footballers with sighted control groups, based on a previous study^28^. Their foot preferences (i.e., the leg preferred to kick a ball) were self-declared; of the 12 participants, 11 were right-footed and the remaining participant was a left-footed nonathlete. The group of “blind footballers” included totally blind and low-vision individuals who played, and regularly trained in blind football. Three players were in B1; two players were in B2; one player was in B3, according to the classification rules of the International Blind Sports Federation^29^. B1 players are those who have a visual acuity lower than LogMAR 2.6, B2 players are those who have visual acuity ranging from LogMAR 1.5–2.6 and/or visual field constricted to a diameter of less than 10 degrees, and B3 players are those who have visual acuity ranging from LogMAR 1.4–1.0 and/or visual field constricted to a diameter of less than 40 degrees. The characteristics of the blind footballers are shown in Table 1. One B1 player lost his vision before the age of 2 years, and two lost their vision between the ages of 20–30 years. One B2 player had congenital visual impairment, and one had visual impairment at the age of 10 years. One B3 player had visual impairment at the age of 12 years. All participants were screened for possible hearing impairments by audiometry. Audiometry was performed with a pure tone audiometer (AA-32W1; Rion Co., Ltd., Tokyo, Japan), and the presented frequencies ranged from 250 to 4000 Hz. Hearing threshold levels were calculated for each ear by averaging the threshold for four frequencies: 500, 1000, 2000, and 4000 Hz. Neither the hearing threshold level at each frequency, nor the average hearing threshold levels of each participant were more than 20 dB. Audiometry was used to confirm that none of the participants had hearing impairment. The present study was conducted in accordance with the Declaration of Helsinki and was approved by the Ethics Committee of the Faculty of Health and Sport Sciences, University of Tsukuba. Informed consent was provided by all participants prior to the experiments.

**Table 1:** Characteristics of blind football players.

Participants	Age	Class	Age at onset of visual impairment
BF1	29	B1	23
BF2	22	B2	10
BF3	22	B3	12
BF4	24	B2	Congenital
BF5	40	B1	28
BF6	27	B1	1.5

### Apparatus and task

**Figure 1:**
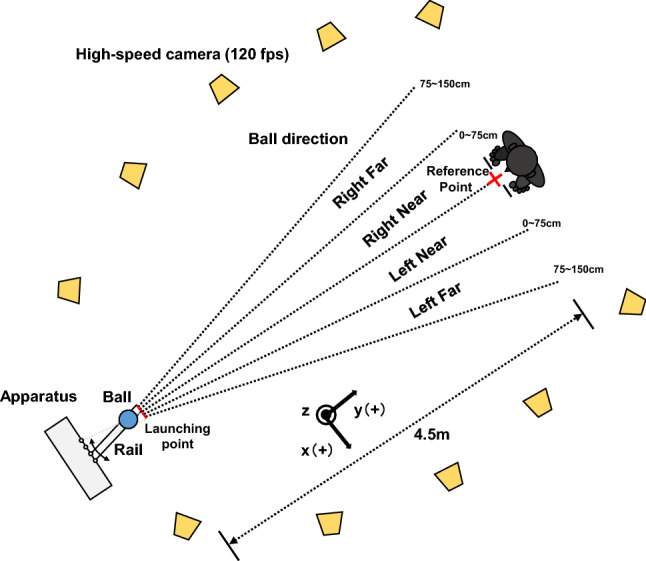
Experimental set-up. Reflective markers were attached to the body of each participant. The three-dimensional position of the reflective markers was computed using an infrared camera motion system with ten cameras. The cameras were placed around the ball and the participant’s body. The ball was launched towards four different directions (right far, right near, left far, and left near). The near space ranged between 0 and 75 cm laterally away from the reference point, and the far space ranged between 75 and 150 cm laterally away from the reference point.

Figure 1 shows a top-down view of the experimental setup. The experiment was conducted in a non-anechoic room (7.3 m × 7.3 m × 2.5 m). The present experiment was reasonably performed under a reverberant listening condition^4,28^, because this reflects an actual condition in playing blind football and therefore has an ecological approach. The floor of the room was covered by joint mats (B0013HF3DS; TOEI LIGHT Co., Ltd., Tokyo, Japan) to prevent the risk of injury by fall. Thin threads were attached to the mat surface to provide the participants standing on the mat with spatial information about the original position. The reference point was defined as the intermediate position between the two threads. An infrared camera motion system with 10 cameras (OptiTrack Flex13, 120 fps; NaturalPoint Inc. Corvallis, OR, USA) was used to compute the three-dimensional position of reflective markers attached to the body of each participant. The cameras were placed around the ball and the participant's body (see Fig. 1), such that we could focus on every infrared reflective marker with at least three cameras at the same time to ensure optimal recordings. Each participant was outfitted with a total of 16 infrared reflective markers (14 mm in diameter). Three infrared reflective markers were arranged on the head to form a triangle, according to a previous study^30^, with the marker on the top of head as the tip. Other reflective markers were attached as follows: One marker on the superior margin of the sternum (breastbone), one marker on each shoulder, each greater trochanter, each lateral epicondyle of femur, each lateral malleolus, each toe, and each heel.

The official ball approved by the Japan Blind Football Association was used in the experiment. The ball, with infrared reflective tape, was launched to the participants using a self-made apparatus, which contained an aluminium rail to adjust the ball speed and direction. The inclination of the rail was 45 degrees to the ground. The ball was released manually by the experimenter from one of three different height positions on the rail, such that the ball speeds were 2.4 m/s, 2.2 m/s, and 1.92 m/s. The distance between the ball launching point and the reference point was 4.5 m. The ball direction (i.e., the angle of the vector from the ball launching point to the ball trapping point relative to the vector from the ball launching point to the reference point) was changed by adjusting the direction of the rail trial-by-trial to prevent the participants from anticipating the ball direction prior to launching. Moreover, there was some noise each time the rail was moved by the experimenter. For this reason, the experimenter intentionally generated the noise by moving the rail for each trial, even when a ball was continuously launched to the same direction as the previous trial, so that we could prevent participants from anticipating the ball direction prior to launching. Four directional conditions were set as shown in Fig. 1 (i.e., right far, right near, left far, and left near). Near conditions ranged from 0 to 75 cm laterally away from the reference point, and far conditions ranged from 75 to 150 cm laterally away from the reference point, so that the ball direction ranged between − 18 and 18 degrees.

The participants were instructed to stand at the original position wearing socks. They were blindfolded with an eye mask during the task to prevent them from gaining knowledge of the experimental setup. The participants performed trapping an approaching ball by using their right foot in the natural condition, without instruction on head movements. They were instructed to trap the ball at their feet as accurately as possible. The participants returned to the original position after ball trapping to confirm that they were ready for the next trial.

### Procedure

The participants performed the ball trapping task on different types of trials, that differed according to the ball speed and direction. A total of 12 practice trials were performed: Three trials for each of the three different speeds (2.4 m/s, 2.2 m/s, and 1.92 m/s), and four different directions (right far, right near, left far, and left near) in order to familiarize themselves with the task. After the practice trials, the participants completed 36 experimental trials (12 trials × 3 blocks). The order of the ball speed and direction was counterbalanced. Participants had a 5-min break between the blocks. All participants completed a total of 48 trials during the experiment.

### Analysis

**Figure 2:**
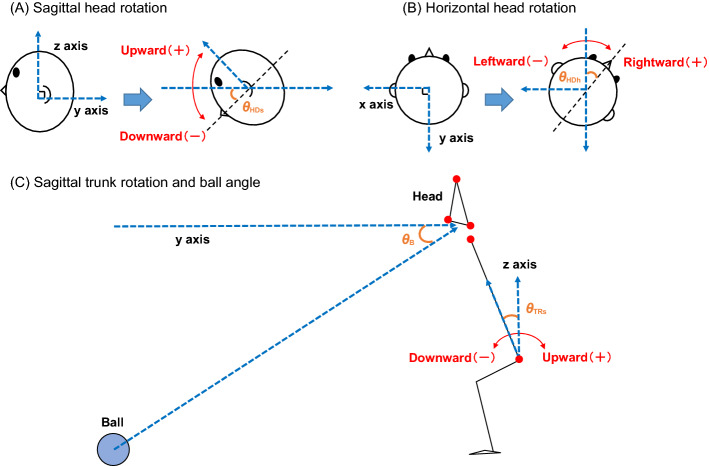
Measurement of head, trunk, and ball angles. (**A**) shows the head rotation angle in the sagittal plane (*θ*_HDs_). Downward and upward head rotations with reference to the z-axis were defined as negative and positive values, respectively. (**B**) shows the head rotation angle in the horizontal plane (*θ*_HDh_). Leftward and rightward head rotations with reference to the x-axis were defined as negative and positive values, respectively. (**C**) shows the trunk rotation angle in the sagittal plane (*θ*_TRs_). Downward and upward trunk rotations with reference to the z-axis were defined as negative and positive values, respectively. (**C**) also shows the ball angle (*θ*_B_); in all cases, the ball angle refers to a negative value, because the ball is directed and launched towards the participant.

Marker position data were low pass filtered with a cut-off frequency of 5 Hz. A custom-written MATLAB code (R2019a, The MathWorks Inc., Natick, MA, USA) was used to calculate the kinematic variables. Some variables related to head and body movements were defined: Head rotation angle in the sagittal plane (i.e., pitch), head rotation angle in the horizontal plane (i.e., yaw), trunk rotation angle in the sagittal plane, and ball angle. The head rotation angle in the sagittal plane (*θ*_HDs_) was derived from two vectors (see Fig. 2A): The vector from the midpoint between the markers on both ears to the top of the head, and the unit vector on the z-axis (vertical direction). Downward and upward head rotations with reference to the z-axis were defined as negative and positive values, respectively. The head rotation angle in the horizontal plane (*θ*_HDh_) was derived from two vectors (see Fig. 2B): The vector from the marker located on the right ear to the marker located on the left ear, and the unit vector on the x-axis (medio-lateral direction). Leftward and rightward head rotations with reference to the x-axis were defined as negative and positive values, respectively. The trunk rotation angle in the sagittal plane (*θ*_TRs_) was derived from two vectors (see Fig. 2C): The vectors from the midpoint of both greater trochanters to the midpoint of both shoulders, and the unit vector on the z-axis. Downward and upward trunk rotations with reference to the z-axis were defined as negative and positive values, respectively. The ball angle (*θ*_B_) was derived from two vectors (see Fig. 2C): The vectors from the ball to the midpoint between markers on both ears, and the unit vector on the y-axis (anterior–posterior direction). In all cases, the ball angle refers to a negative value, since the ball was directed and launched towards the participant.

To calculate the variables at different time points, we defined the time of ball launching as the first frame when the ball bounced to the floor. The time of ball trapping was defined as the first frame when the ball made contact with the participants’ right foot. In the absence of ball contact, the time of ball trapping was defined as the first frame when the distance between the ball and the midpoint between the toe and heel on the right foot was below 0.20 m on the y-axis. This was because the total distance calculated by adding the radius of the ball and half the width of right foot was approximately 0.20 m on the axis if the right foot contacted the ball.

**Figure 3:**
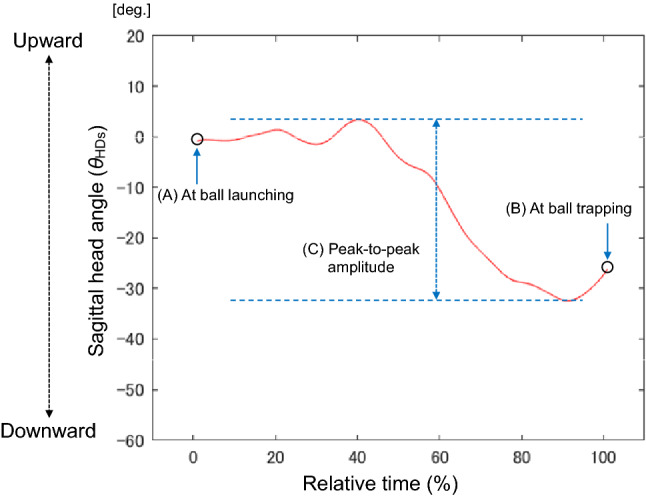
Illustration of rotation in the sagittal plane and the calculation of each angle: (**A**) the head angle at ball launching, (**B**) the head angle at ball trapping, and (**C**) the peak-to-peak amplitude.

Kinematic variables related to head and trunk rotations included the angle at the time of ball launching (Fig. 3A), the angle at the time of ball trapping (Fig. 3B), and the peak-to-peak amplitude (Fig. 3C) calculated by the difference between the maximum and minimum angles from the time of ball launching to the time of ball trapping.

In order to explore the relationship between the head direction and the ball position during ball trapping, the coefficient of correlation value between the angle of head rotation in the sagittal plane and the ball angle was measured as variable of head-ball coupling. As a valid analysis for head-ball coupling, our study applied the method of correlation analysis, which was used to reveal a correlation between head directions and bat positions at the time of bat-ball contact in the previous study^24^. The head and ball movement trajectory for each trial was normalized to 101 time points (1–101); this was accomplished by resampling the time of each trajectory using the interp1 function of MATLAB. The head rotation angles in the sagittal plane relative to those at the time of ball launching were also calculated for each 10% time point.

With regards to performance variables, the absolute error (AE) and variable error (VE) were calculated from the distances between the centre of the ball and the midpoint between the toe and the heel on the right foot at the ball trapping point. If the right foot did not make contact with the ball, that is, the ball passed by the participant’s foot, the error value was calculated based on the local minimum between the distances on the x-axis. AE was defined as an absolute value of the distance between the centre of the ball, and the midpoint between the toe and the heel on the right foot on the x-axis. VE was defined as the standard deviation of the constant error, which was calculated by the distance between the centre of the ball and the midpoint between the toe and the heel on the right foot on the x-axis. These performance variables of the ball trapping task did not seem to be affected by footedness (i.e., the skill of the foot movements). In fact, the left-footed participant's performance within the sighted group was 3rd and 4th in AE and VE, respectively. It was clear that a significant negative result was not found in the participant; the data were therefore treated in the same way as for the other participants.

The Shapiro–Wilk test was used to assess whether the data were normally distributed (*p* > 0.050) based on the analysis performed by the Finocchietti et al.^28^, and Levene's test was used to assess the homogeneity of variance (*p* > 0.050). A two-tailed Student’s t-test was used to compare the variables between blind footballers and sighted nonathletes. The nonparametric test (two-tailed Mann–Whitney U-test) was used when the assumption of normality was violated. A repeated measures ANOVA (3 blocks) was used to confirm whether practice effects led to improvements in task performance through 3 blocks (36 trials) in the sighted nonathletes who were not familiar with performing the ball trapping task. No significant main effects of the block were confirmed either on AE or VE; therefore, the data of 36 trials in the AE and the VE of the sighted nonathletes were combined and analysed. Data of one trial from two sighted nonathletes were excluded from the actual analysis as they were not recorded correctly. Analysis was conducted using IBM SPSS Statistics software (ver. 24.0; IBM Corp., Armonk, NY, USA). The alpha level was set at 0.05.

## Results

### Performance

**Figure 4:**
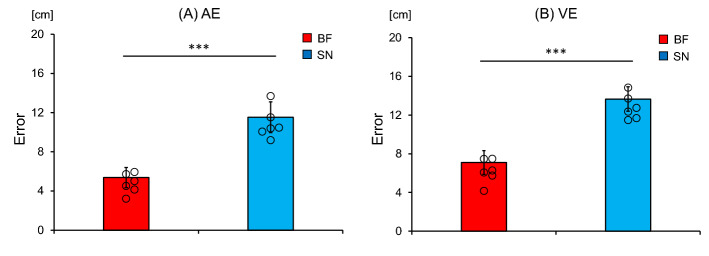
Absolute error (AE) and variable error (VE). Comparison of the mean (**A**) AE and (**B**) VE of the blind footballers (BF) and the sighted nonathletes (SN). The circles represent the mean AE and VE of each individual. The bars represent the standard deviation. ***p < 0.001.

The AE of the blind footballers (5.37 ± 1.02 cm) was significantly smaller (t [10] = 8.04, *p* < 0.001) than that of the sighted nonathletes (11.52 ± 1.57 cm), as shown in Fig. 4A. Furthermore, the VE of the blind footballers (7.1 ± 1.23 cm) was significantly smaller (t [10] = 9.11, *p* < 0.001) than that of the sighted nonathletes (13.66 ± 1.27 cm), as shown in Fig. 4B.

### Head rotation angle in the sagittal plane

**Figure 5:**
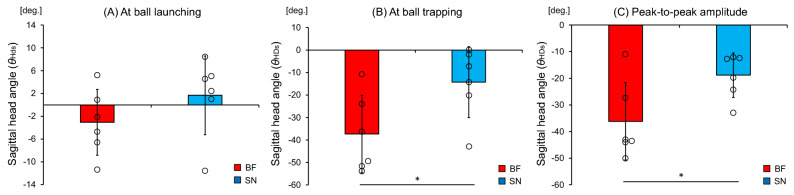
Mean head rotation angles in the sagittal plane. Comparison of the mean (**A**) head angle at the time of ball launching, (**B**) head angle at the time of ball trapping, and (**C**) peak-to-peak amplitude between the blind footballers (BF) and the sighted nonathletes (SN). The circles represent the mean head angle of each individual. The bars represent the standard deviation. *p < 0.05.

With regards to the head rotation in the sagittal plane, there was no significant difference in the head angle at the time of ball launching (t [10] = 1.29, *p* = 0.227) between the blind footballers (− 3.1 ± 5.8 deg.) and the sighted nonathletes (1.7 ± 6.9 deg), as shown in Fig. 5A. However, there was a significant difference in the head angle at the time of ball trapping (t [10] = 2.42, *p* = 0.036) between the blind footballers (− 37.3 ± 17.2 deg.) and the sighted nonathletes (− 14.3 ± 15.8 deg.), as shown in Fig. 5B; these results indicated that the blind footballers directed their head more downward than the sighted nonathletes. There was also a significant difference in the peak-to-peak amplitude of the head angle (t [10] = 2.53, *p* = 0.030) between the blind footballers (− 36.2 ± 14.5 deg.) and the sighted nonathletes (− 18.8 ± 8.4 deg.), as shown in Fig. 5C; these results indicated that the blind footballers showed a larger downward head rotation than the sighted nonathletes.

### Head-ball coupling

**Figure 6:**
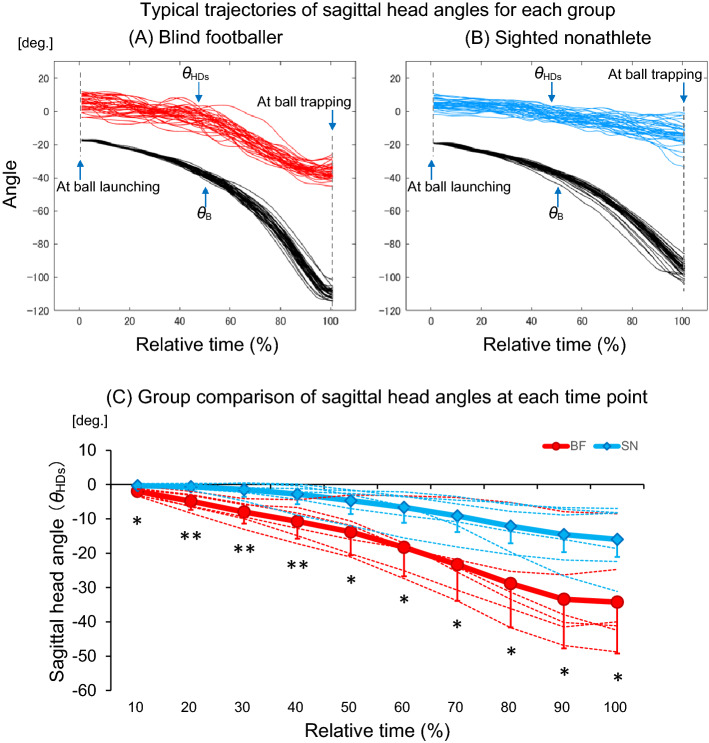
Trajectories of the head rotation angles in the sagittal plane at each time point. Demonstration of the trajectories of the head rotation angles in the sagittal plane and ball angles for (**A**) one blind footballer and (**B**) one sighted nonathlete. The trajectories of the head rotation angles represent the approximate mean values of each group. (**C**) Comparison of the relative angles of head rotations in the sagittal plane at each time point between the blind footballers (BF) and the sighted nonathletes (SN). The red and blue coloured bold lines represent the mean values for each group. The red and blue coloured thin dotted lines represent the values of the individuals for each group, and the bars represent the standard deviation. *p < 0.05, **p < 0.01.

Figure 6 shows the trajectories of the head rotation angles in the sagittal plane at each time point. Figure 6A,B show the trajectories of all trials for one representative in the blind footballers and the sighted nonathletes, respectively. Although strong correlations were observed between the head rotation angle in the sagittal plane and the ball angle in both groups (blind footballers: r = 0.95 [range: 0.83–0.97], sighted nonathletes: r = 0.88 [range: 0.56–0.95]), there was no significant difference in the mean of the coefficient of correlation between the groups (U = 9, Z = − 1.441, *p* = 0.150). Furthermore, the relative angles of the head rotations in the sagittal plane at each time point were significantly larger in the blind footballers than those in the sighted nonathletes at all time points (Fig. 6C). These results indicated that more downward head rotations were consistently observed in the blind footballers (10%: − 2.0 ± 1.2 deg., 20%: − 4.8 ± 2.5 deg., 30%: − 8.0 ± 3.4 deg., 40%: − 10.7 ± 5.0 deg., 50%: − 13.8 ± 6.8 deg., 60%: − 18.3 ± 8.4 deg., 70%: − 23.3 ± 10.6 deg., 80%: − 28.9 ± 12.8 deg., 90%: − 33.4 ± 14.2 deg., and 100%: − 34.3 ± 14.9 deg.), when compared to the sighted nonathletes (10%: − 0.3 ± 0.4 deg., 20%: − 0.6 ± 0.8 deg., 30%: − 1.5 ± 2.0 deg., 40%: − 2.8 ± 3.4 deg., 50%: − 4.7 ± 4.1 deg., 60%: − 6.7 ± 5.0 deg., 70%: − 9.1 ± 5.6 deg., 80%: − 12.2 ± 6.8 deg., 90%: − 14.6 ± 8.4 deg., and 100%: − 16.0 ± 9.8 deg.).

### Trunk rotation angle in the sagittal plane

**Figure 7:**
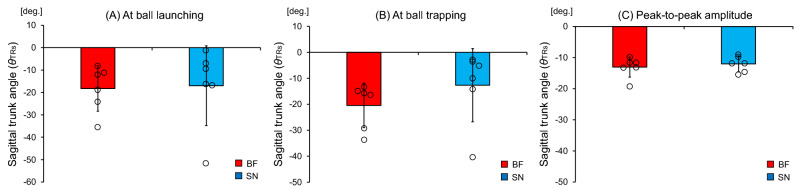
Mean trunk rotation angles in the sagittal plane. Comparison of the mean (**A**) trunk angle at the time of ball launching, (**B**) trunk angle at the time of ball trapping, and (**C**) peak-to-peak amplitude between the blind footballers (BF) and the sighted nonathletes (SN). The circles represent the mean trunk angles of each individual. The bars represent the standard deviation.

With regards to the trunk rotation in the sagittal plane, there was no significant difference in the trunk angle at the time of ball launching (t [10] = 0.15, *p* = 0.883) between the blind footballers (− 18.2 ± 10.1 deg.) and the sighted nonathletes (− 17.0 ± 17.8 deg.), as shown in Fig. 7A. Furthermore, there was no significant difference in the trunk angle at the time of ball trapping (t [10] = 1.16, *p* = 0.275) between the blind footballers (− 20.4 ± 8.6 deg.) and the sighted nonathletes (− 12.7 ± 14.1 deg.), as shown in Fig. 7B. There was no significant difference in the peak-to-peak amplitude of trunk angle (t [10] = 0.61, *p* = 0.554) between the blind footballers (− 13.0 ± 3.2 deg.) and the sighted nonathletes (− 12.0 ± 2.5 deg.), as shown in Fig. 7C.

### Head rotation angle in the horizontal plane

**Figure 8:**
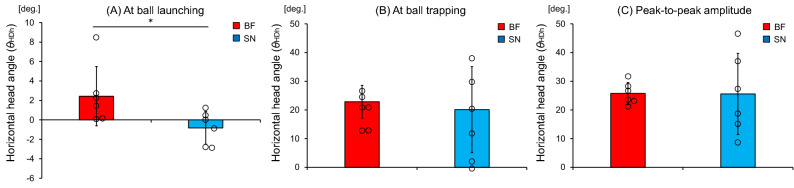
Mean head rotation angles in the horizontal plane. Comparison of the mean (**A**) head angle at the time of ball launching, (**B**) head angle at the time of ball trapping, and (**C**) peak-to-peak amplitude between the blind footballers (BF) and the sighted nonathletes (SN). The circles represent the mean head angles of each individual. The bars represent the standard deviation. *p < 0.05.

As for the head rotation in the horizontal plane, there was a significant difference in the head angle at the time of ball launching (t [10] = 2.29, *p* = 0.045) between the blind footballers (2.4 ± 3.0 deg.) and the sighted nonathletes (− 0.8 ± 1.7 deg.), as shown in Fig. 8A; these results indicated that the blind footballers directed their head more rightward than did the sighted nonathletes. In contrast, there was no significant difference in the head angle at the time of ball trapping (t [6.41] = 0.42, *p* = 0.689) between the blind footballers (22.8 ± 5.7 deg.) and the sighted nonathletes (20.1 ± 15.0 deg.), as shown in Fig. 8B. Moreover, there was no significant difference in the peak-to-peak amplitude of the head angle (t [5.74] = 0.03, *p* = 0.979) between the blind footballers (25.7 ± 3.8 deg.) and the sighted nonathletes (25.6 ± 14.1 deg.), as shown in Fig. 8C.

## Discussion

The purpose of the present study was to investigate the strategy of blind footballers when localizing a dynamic sound source, such as a moving ball. In particular, we focused on the characteristics of head rotations in blind footballers compared to sighted nonathletes when trapping an approaching ball. The error of ball trapping in the blind footballers was smaller than that of the sighted nonathletes. Furthermore, the downward head rotation angle of the blind footballers was larger than that of the sighted nonathletes at the time of ball trapping, but not at the time of ball launching. The relative angle of downward head rotation of the blind footballers was significantly larger than that of the sighted nonathletes from an early point after ball launching to the moment of ball trapping.

Importantly, we observed significantly larger downward head rotations in the blind footballers than in the sighted nonathletes, as shown by the group differences in the angle at the time of ball trapping, as well as the peak-to-peak amplitude. There are some possible explanations for these results. Firstly, the large downward head rotations observed in the blind footballers in the present experiment seem to be important to enable participants to use dynamic cues provided by the head rotations, and to localize the approaching ball in front of the head. Indeed, dynamic cues provided by head rotations have been shown to improve sound localization accuracy^9,12,13^. It is reasonable to assume that the blind footballers used dynamic cues provided by the head rotations for accurate localization of the ball location. Secondly, previous studies have shown that sound localization is accurate when sound is presented in the frontal space^31,32^, especially in front of the listeners’ heads^17–19^ rather than in peripheral locations. These previous findings support the idea that localizing a sound in front of the head with downward head rotation is an effective strategy to accurately localize the sound of the ball approaching participant’s foot. Moreover, it is probable that sighted individuals visually ‘capture’ the location of the sound source without completing the head turning when searching for the auditory target. In other words, due to the free movement of the eyes, there is no need for sighted individuals to complete the turning movements of the head in order to accurately localize the sound source by vision^33–35^. In contrast, blind footballers such as blind individuals in general, cannot use this strategy, and therefore must indeed turn the head in the direction of the sound source in order to accurately localize it. Taken together, the present results suggest that blind footballers use a larger downward head rotation to localize the ball more directly in the front of the head when sound is localized accurately.

A previous study has suggested that the forward leaning of the trunk observed in blind footballers could be used as a strategy to maximize the quality of auditory inputs to guide postural control during passing/receiving a ball^28^. However, in this previous study, the kinematic movement patterns of the head and the trunk when receiving the ball were not confirmed. The present study demonstrated no significant differences between the blind footballers and the sighted nonathletes with regards to all parameters related to the trunk rotation angles in the sagittal plane. The difference between the present findings and those of Finocchietti et al.^28^ may be due to differences in the characteristics of the task. For instance, in the previous study, ball passing against a wall (i.e., a sequence of kicking and receiving the ball) was the main task, wherein the trunk flexion angle was extracted from data averaged between − 250 and 250 ms around the foot contact with the ball. For the reason, it is possible that the forward tilt of the trunk observed in the blind footballers may be attributed to a specific movement to kick the ball, rather than localize it. In contrast, receiving the ball was the main task in the current study; thus, localization of the ball was necessary, and it seems that the blind footballers used a strategy to rotate the head towards the ball in order to accurately localize it. The present study has added a new finding to this field, in that the strategy of blind footballers to accurately localize a ball in trapping is not attributed to the forward tilt of the trunk, but rather to the larger downward head rotation. The strategy observed in the present study could be specific to the ball trapping task. It is expected that further studies will lead to a greater understanding of a strategy for sound localization in other auditory–motor tasks related to the actual playing of blind football.

A previous finding has suggested that blind footballers can develop specific strategies to localize a dynamic sound to maximize the quality of auditory inputs, due to long-term training in blind football^28^. More than just localizing the sound source, blind footballers can also develop strategies for interacting with that sound source through their training in blind football. Indeed, it is possible to assume that the present task is not only about localizing a sound, but also to interact with the source of sound. This interaction affects the manner that the players perceive the sound and its location. This assumption is in accordance with the common coding approach to perception and action^35–38^ and with the theory of event coding^39^. According to these approaches, perceived events and planned actions share a common representational domain, and therefore, the manner one perceives events is influenced by his/her typical or intended interaction with them. From these findings, it seems that blind athletes can use a strategy to couple the perception of a dynamic sound source and the planned action for trapping. Thus, the planning of ball trapping should indeed be different between the blind footballers and the sighted nonathletes, not only because the blind footballers are thought to be more accurate in localizing the sound source, but also because their typical interaction with that sound source differs from that of the sighted nonathletes. For these reasons, it is reasonable to explain that the large downward head rotation of the blind footballers is due to the experience of playing blind football.

With regards to the auditory–motor performance of ball trapping, the blind footballers showed a better performance compared to that of the sighted nonathletes. The accuracy of auditory–motor performance, such as reaching and pointing to auditory targets, has been shown to be influenced by the position of the sound source in relation to the head^20,21^. Pointing accuracy to auditory targets was higher under the long sound duration condition, in which listeners showed a larger amplitude of head movements (i.e., range of motion) and a larger final heading angle towards targets^21^. The authors suggested that the accuracy of pointing to long stimuli could be due to the contribution of heading towards the target, thereby providing a more accurate frame of reference for the anticipated control of pointing. They also suggest that the heading direction is coded in a body-centred reference frame, and can be used directly by the reaching motor command that shares the same reference frame; the results of the present study are in agreement with those of these previous studies. The fact that the sighted nonathletes were blindfolded during the task could lead to an awkward or inefficient posture and worse auditory–motor performance. A previous study compared the postural control of blind footballers to sighted footballers and sighted individuals during unstable standing balance tasks under closed-eyes conditions, showing no difference of the balance performance between the blind footballers and the other groups before and after training^40^. Therefore, it seems that the lower performance in the sighted nonathletes did not necessarily result from an awkward or inefficient posture due to the blindfolded condition. Our results indicate that precise ball trapping in blind footballers can be not only due to the accurate frame of reference by head rotation towards the ball, but also the head-centred reference frame that is similarly shared and used by the motor control of the foot in ball trapping.

No significant difference in the groups was found regarding the coefficient of correlation value between the head rotation angle and the ball angle; this demonstrates that both groups rotated their heads downward according to the approaching ball. However, the relative angle of the downward head rotations in the sagittal plane at each time point was significantly larger in the blind footballers than in the sighted nonathletes from the early time point after ball launching to the moment of ball trapping. These results may indicate that the blind footballers kept the ball in a more consistent direction relative to the head throughout the ball trapping phase. Previous studies report a strategy of head-ball coupling that couples the head direction to the movement of the ball to track the target in skilled batters^22–25^. For instance, elite baseball batters have been found to rotate their heads until bat-ball contact, indicating the possibility that they made head rotation according to the prediction of ball trajectories^24^. Elite cricket batters have also been found to have a tighter coupling between the ball and the head, such that the ball was kept in a consistent egocentric direction throughout batting^22^. The study by Mann et al.^22^ indicates the functional advantage of this behaviour to keep the target in a consistent direction relative to the head, since visuo–motor tasks, like catching and hitting, are understood to be controlled in an egocentric manner. These findings also highlight the importance of keeping the ball in a consistent egocentric direction relative to the head in the auditory–motor task, which requires the prediction of ball trajectories and interception of the ball. Spatial location of an object is, in principle, coded with an egocentric and allocentric reference of frames^41^. Though sound location can be represented in multiple coordinate frames^42,43^, it is mainly coded in a single head-centred reference frame^26,27^ that is processed based on auditory cues, such as interaural level and time difference cues, and monaural spectral cues^44^. These previous findings can explain why the blind footballers continued to rotate their head to keep the ball in a consistent head-centred egocentric direction in tracking the approaching target, in terms of its localization on the basis of auditory cues. Therefore, our study suggests that blind footballers couple the downward head rotation with the movement of an approaching ball, while ensuring that the ball was kept in a consistent egocentric direction relative to the head throughout ball trapping.

The head rotation angle in the horizontal plane at the time of ball launching was slightly, but significantly larger in the blind footballers than that in the sighted nonathletes. Differences in the timing and intensity of sounds arriving at the two ears are well-documented cues that can be used to judge the direction of the sound source^45^. In general, binaural cues, like interaural time and interaural level differences, are mentioned as the primary cues for sound localization in the horizontal plane^5,46^. Even though the ball direction was random in each trial in the present study, the initial point of the ball launching remained constant. It may be possible that the blind footballers could prepare for quick prediction of the ball trajectory based on auditory cues provided by the slightly increased horizontal head rotation angle at the time of ball launching.

Various studies have reported that blind individuals have supra-normal auditory abilities in comparison to sighted individuals regarding sound localization^47–49^, pitch discrimination^50–52^, and sound motion^53^, indicating that the abilities of blind individuals are assumed to be attributed to compensating for visual loss. Recent findings suggest that rapid and accurate identification of sound direction in blind footballers can be due to long-term training in blind football^3,4,8^. Furthermore, Velten et al.^3,4^ reported that blind footballers are more precise than blind individuals in terms of the identification of sound direction; this indicates that the results are not solely due to the absence of vision and enhancement in auditory perception accuracy, but are also related to the practice of blind football. Regarding the years of blind football playing experience, Mieda et al.^8^ demonstrated that the more experienced group showed a tendency to identify sound direction more rapidly than did the less experienced group. Thus, their results indicate that rapid identification of sound direction in blind footballers can be attributed to a long period of blind football playing experience. Taken together, it is reasonable to assume that the long-term experience of blind football, but not the visual impairments, could enable the blind footballers to localize the ball more accurately than the sighted nonathletes.

Despite the present findings, the question remains whether the large downward head rotation adopted by the blind footballers was due to the lack of vision. If this is the case, blind nonathletes should present a similar pattern, even with differences in the performance of ball trapping. One possibility for this is that blind individuals may focus on self-induced motion of the surrounding sound sources resulting from active head and/or body movements much more intensely than sighted individuals to utilize the reliable information about the deviation between perceived and physical sound-source locations^53^. Another possibility which may be considered is that blind individuals use the egocentric frame of reference due to the loss of vision when localizing a sound^54,55^, which could result in keeping a moving ball in a consistent egocentric direction relative to the head. Future studies could address this question by clarifying the characteristics of head rotation of blind footballers compared with blind individuals during ball trapping.

## Conclusions

The present study demonstrated a higher performance of ball trapping and a larger angle of downward head rotation in the blind footballers compared to the sighted nonathletes. However, no significant difference in trunk rotation angle was found between the groups. These results suggest that blind footballers use a larger downward head rotation as a strategy for precise ball trapping, ensuring localization of the ball towards the front of the head. These results also suggest that the strategy of blind footballers to accurately localize a ball in trapping is not attributed to forward tilt of the trunk, but rather to downward rotation of the head. The relative angle of the downward head rotation of the blind footballers was consistently larger than that of the sighted nonathletes from an early time point after ball launching to the moment of ball trapping. Our study suggests that blind footballers couple the downward head rotation with the movement of an approaching ball, ensuring the ball is kept in a consistent egocentric direction relative to the head throughout ball trapping.
